# Decoding the association between health level and human settlements environment: a machine learning-driven provincial analysis in China

**DOI:** 10.3389/fpubh.2025.1672479

**Published:** 2025-09-03

**Authors:** Haidong Zhu, Xiaoqing Peng

**Affiliations:** Department of Architecture and Civil Engineering, City University of Hong Kong, Hong Kong, Hong Kong SAR, China

**Keywords:** health level, human settlement environment, machine learning, XGBoost, Shapley additive explanations

## Abstract

**Background:**

Rapid urbanization in China has significantly reshaped the human settlement environment (HSE), bringing opportunities and challenges for public health. While existing studies have explored environmental-health relationships, most are confined to micro-level contexts, focus on single environmental dimensions, or assess specific diseases, thus lacking a comprehensive, macro-level understanding.

**Objective:**

This study aims to assess the associations between population health level and multidimensional HSE features at the provincial level in China and uncover nonlinear relationships and interaction effects underlying the association between HSE and population health level.

**Methods:**

Using panel data from 31 Chinese provinces spanning 2012 to 2022, a composite Health Level Index (HLI) was constructed based on four core health indicators using the Entropy-TOPSIS method. 19 HSE indicators covering five dimensions—ecological environment, living environment, infrastructure, public services, and sustainable environment—were selected as explanatory variables. The study employed the XGBoost machine learning algorithm to model the relationship between HSE and HLI. SHAP values and Partial Dependence Plots (PDPs) were used to interpret feature importance, nonlinear relationships, threshold values, and interaction effects.

**Results:**

XGBoost outperformed all benchmark models, confirming its strong predictive capacity. SHAP analysis identified six key features—number of medical institution beds (NMIB), urbanization rate (UR), mobile phone penetration rate (MPPR), road area per capita (RAPC), population density (PD), and urban gas penetration rate (UGPR)—as the most influential factors. Nonlinear relationships and threshold effects were observed between key features and population health level. PDP plots further revealed that optimal health levels are typically associated with high UR, high MPPR, high RAPC, and moderate NMIB, underscoring the importance of structural synergy over isolated infrastructure expansion.

**Conclusion:**

This study provides robust evidence that the relationship between HSE and health is nonlinear, multidimensional, and highly interactive. Effective urban health governance requires coordinated development of urbanization, digital infrastructure, and public services, along with rational healthcare resource allocation. The findings offer actionable insights for health-oriented urban planning and policy formulation in rapidly urbanizing regions.

## 1 Introduction

Urbanization has reshaped the global HSE, especially in fast-growing economies such as China. As the world’s largest developing country, China’s urbanization level has risen rapidly over the decades, with a total of 694 cities at the end of 2023, compared to 129 cities at the start of the new China, according to the National Bureau of Statistics of China. By the end of 2023, the urbanization rate of China’s resident population was 66.16%, compared to 10.64% at the end of 1949 (1).

The HSE refers to the living spaces inhabited by human populations. It is a geographical space closely associated with human survival and serves as a primary arena where humans utilize and transform nature (2). From a research perspective, the HSE is a multidimensional system, and the specific dimensions involved often vary depending on the research perspective. For example, some studies have evaluated the suitability of human settlements by integrating factors such as economic vitality, public services, infrastructure, topography, and climate conditions (3–5). Other scholars have focused on sustainability by incorporating elements like transportation, cultural resources, and living conditions into their assessments (6, 7). Additionally, certain studies have assessed the vulnerability of human settlements through dimensions such as the natural environment, social environment, and residential conditions (8, 9). At present, there is no universally agreed-upon set of evaluation indicators for human settlements; instead, indicator selection is typically determined by the specific research objectives, theoretical framework, and data availability.

At the same time of rapid urbanization, the contradiction between HSE and residents’ health is becoming more and more prominent. It has been shown that large-scale urban expansion has led to the shrinkage of green space (10), the intensification of the heat island effect (11), and the spread of air and water pollution (12, 13). These changes directly or indirectly impact the physical or mental health of the population, for example, leading to an increase in the incidence of respiratory diseases, chronic diseases (14–16), and psychological problems such as anxiety and depression, among others (17). Assessing the extent to which the HSE is associated with population health level and exploring the predictive relationships between them is becoming a hotspot of interest in the fields of health, environment, and urban studies.

Existing researches on environment and health risk is abundant, but in terms of research scale, are mainly focus on community, urban environment (18, 19), lack of research in macro scale such as provincial, national, and so on, and in terms of selection of environmental factors, are mainly focus on single social environment, natural and built environments (20–22), lack of comprehensive exploration of multidimensional environmental factors, and in terms of health impacts, are limited to focusing on the environmental impacts on the risks of a specific disease (23), and lack of focus on the overall health of the population. To address these gaps, this study aims to examine the relationship between multidimensional HSE factors and the overall health level of the population at the provincial scale in China, using interpretable machine learning techniques to uncover nonlinear relationships and interaction effects.

## 2 Data and methods

### 2.1 Data

The dataset of this paper is divided into two parts. In the first part, referring to previous studies (24–26), four indicators were selected to represent the health level of the population: incidence of class A and B notifiable infectious diseases, mortality of class A and B notifiable infectious diseases, human mortality, and average life expectancy. In China, notifiable infectious diseases are categorized into three classes—A, B, and C—according to their potential threat to public health, with severity decreasing from A to C; classes A and B, which together encompass 29 diseases (2 in class A and 27 in class B), include the most serious infectious diseases and are therefore widely adopted as core measures of population disease burden (27). Together with human mortality, these disease-related indicators capture both the prevalence and fatality of major health threats, while average life expectancy provides a broader perspective on long-term population well-being, reflecting the cumulative effects of healthcare quality, living conditions, and social development. This combination of short-term disease burden and long-term health outcomes has been extensively used in health evaluation studies (28), and the data are consistently available from official statistical sources, ensuring reliability and comparability across regions. Based on these indicators, the Entropy–TOPSIS method was applied to construct the HLI, which integrates multiple dimensions of health into a single, comprehensive measure. Details of the four selected indicators are presented in Table 1. In the second part, according to the needs of the study, partly referring to the indicators used in previous studies (29–31), the HSE was divided into five dimensions: ecological environment, living environment, infrastructure conditions, public service, and sustainable environment, and 19 secondary indicators were selected. All HSE data can be directly obtained from the aforementioned public sources, except for four indicators—RAPC, number of sanitation vehicles per 10,000 population (NSV), PD, and UR—which were derived through calculation. The calculation methods for these four indicators are presented in Equations 1–4. The details of HSE indicators are shown in Table 2.

**Table 1:** Indicators related to the level of health of the population.

Indicator	Unit	Indicator Attribute
Incidence of Class A and B notifiable infectious diseases	1/100,000	−
Mortality of Class A and B notifiable infectious diseases	1/100,000	−
Human mortality	%	−
Average life expectancy	Years	+

**Table 2:** HSE indicator system.

Primary indicator	Secondary indicator	Unit
Ecological environment	*Per capita* park green area (PCPGA)	*m^2^*
	Chemical oxygen demand emissions (CODE)	10^4^ tons
	Sulfur dioxide emissions (SO₂ Emissions)	10^4^ tons
	Number of sanitation vehicles per 10,000 population (NSV)	Units
Living environment	Urban water penetration rate (UWPR)	%
	Urban gas penetration rate (UGPR)	%
	Population density (PD)	Persons/*km^2^*
	Urbanization rate (UR)	%
Infrastructure conditions	Number of public toilets per 10,000 people (NPT)	Units
	Road area per capita (RAPC)	*m^2^*
	Number of public transportation vehicles per 10,000 People(NPTV)	Standard units
	Mobile phone penetration rate (MPPR)	Units/100 persons
Public service	Number of higher education students per 100,000 people (NHES)	Persons
	Public library floor area per 10,000 population (PLFA)	*m^2^*
	Number of medical institution beds per 10,000 People (NMIB)	Units
	Population served per postal service outlet (PSPSO)	10^4^ persons
Sustainable environment	Daily urban sewage treatment capacity (DUSTC)	10^4^ *m^3^*
	Local financial environmental protection expenditure (LFEPE)	10^8^ yuan
	*Per capita* daily domestic water consumption (PCDDWC)	Liters

The first part of the data comes from the China Health Statistics Yearbook 2013–2023, where the missing years of average life expectancy are filled in by linear interpolation, a method chosen based on relevant studies supporting the linear growth trend of life expectancy (32, 33), thus ensuring the scientific validity and completeness of the data. In total, 279 provincial-level data points on average life expectancy were supplemented. The second part of the data was obtained from the National Bureau of Statistics of China, China Statistical Yearbook 2013–2023, and Statistical Bulletins of Chinese provinces. The final dataset consists of these two parts of data, covering 31 provinces in China for the period 2012–2022. This yields a balanced panel of 341 province-year observations, with a total of 6,820 variable observations used in the analysis. The descriptive statistics of the variables are shown in Table 3.


(1) $$\text{RAPC}=\frac{\text{Total Road Area}}{\text{Resident Population}\;\mathrm{at}\;\text{Year}-\mathrm{End}}$$

(2) $$\mathrm{NSV}=\frac{\text{Number of Sanitation Vehicles}}{\text{Resident Population}\;\mathrm{at}\;\text{Year}-\mathrm{End}}\times 10,000$$

(3) $$\begin{array}{c} \mathrm{PD}=\frac{\text{Resident Population}\;\mathrm{at}\;\text{Year}-\mathrm{End}}{\text{Provincial Administrative Area}} \end{array}$$

(4) $$\begin{array}{c} \mathrm{UR}=\frac{\text{Urban Population}}{\text{Resident Population}\;\mathrm{at}\;\text{Year}-\mathrm{End}} \end{array}$$

**Table 3:** Descriptive statistics of variables.

Variable	Sample size	Mean	Std. dev.	Minimum	Maximum
PCPGA	341	13.47	2.84	5.85	22.84
CODE	341	56.85	49.59	1.76	192.12
SO₂ emissions	341	33.05	35.35	0.11	174.88
NSV	341	1.95	1.64	0.12	15.02
UWPR	341	97.87	3.03	67.57	100.00
UGPR	341	93.78	9.22	29.79	100.00
PD	341	460.36	701.53	2.57	3925.87
UR	341	59.80	12.68	22.86	89.58
NPT	341	3.18	1.26	0.77	9.35
RAPC	341	5.97	2.29	1.14	13.71
NPTV	341	12.73	3.01	5.63	26.55
MPPR	341	104.63	24.07	57.30	189.46
NHES	341	2774.84	855.97	1133.00	5534.00
PLFA	341	118.51	48.23	45.66	335.80
NMIB	341	56.51	11.35	27.15	84.31
PSPSO	341	0.72	0.44	0.15	2.77
DUSTC	341	573.09	497.78	5.00	2971.30
LFEPE	341	155.81	100.91	17.21	747.44
PCDDWC	341	174.24	49.19	91.12	403.62
HLI	341	0.60	0.11	0.36	0.89

### 2.2 Method

#### 2.2.1 HLI construction based on entropy-TOPSIS method

**Figure 1:**
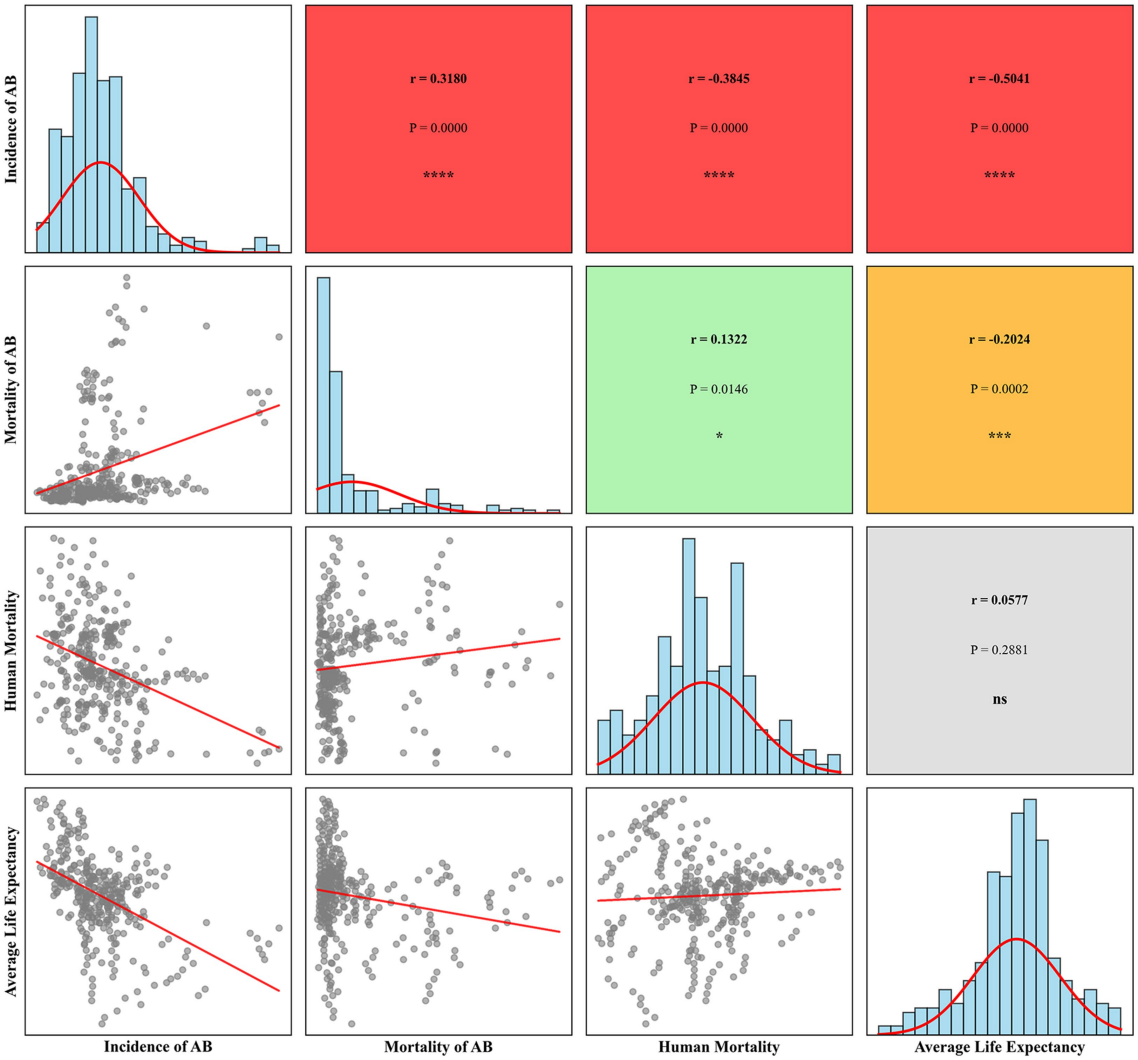
Correlation coefficient matrix of health indicators.

Various evaluation models, such as Fuzzy Comprehensive Evaluation (34), the Analytical Hierarchy Process (AHP) (35), and the Technique for Order of Preference by Similarity to Ideal Solution (TOPSIS) (36), have been utilized in recent studies. To scientifically quantify the population health level in each province in China, this study introduces the entropy weight method combined with the TOPSIS evaluation model to construct the HLI. While standard statistical techniques like Principal Component Analysis (PCA) or Factor Analysis could create weighted composites based on covariance structures (37), our study employs the Entropy-TOPSIS approach for several key advantages: 1. The entropy weight method objectively determines indicator weights based on information content rather than subjective expert judgment (38), avoiding potential bias inherent in AHP or equal weighting schemes; 2. Unlike PCA, which may lose interpretability through linear combinations of variables, entropy weighting preserves the original meaning of each health indicator; 3. TOPSIS provides intuitive relative rankings by measuring proximity to ideal solutions (39), making results more accessible for policy interpretation compared to factor scores; 4. The entropy weight method is used to avoid subjective bias, and the TOPSIS method is used to measure the relative closeness of the samples to the ideal solution, resulting in the formation of the HLI, which is both scientific and comparable. Prior to constructing the composite index, a correlation analysis was conducted on the four variables to ensure their appropriateness for inclusion, with the results presented in Figure 1. The results showed that the absolute values of the correlation coefficients between the variables were all below 0.6, indicating no severe multicollinearity and supporting their suitability for constructing the composite index. The specific steps of the Entropy-TOPSIS method are as follows:

Step 1: Matrix construction of raw data.

Assuming the research object contains m samples ( i=1,2,⋯,m ), 4 core health indicators (Table 1) are selected to form the evaluation system, and the raw data matrix X is defined as:


(5) $$\begin{array}{c} X=[\begin{array}{cccc} x_{11} & x_{12} & x_{13} & x_{14}\\ x_{21} & x_{22} & x_{23} & x_{24}\\ \vdots & \vdots & \vdots & \vdots \\ x_{m1} & x_{m2} & x_{m3} & x_{m4} \end{array}] \end{array}$$

Where: $x_{\mathrm{ij}}$ denotes the raw value of the j-th indicator for the i-th sample.

Step 2: Data standardization.

To ensure comparability and preserve the directional meaning of each indicator, positive and negative indicators are normalized separately.

(1) For positive indicators:


(6) $$\begin{array}{c} x_{\mathrm{ij}}^{\prime }=\frac{x_{\mathrm{ij}}-\min(x_{j})}{\max(x_{j})-\min(x_{j})} \end{array}$$

(2) For negative indicators:


(7) $$\begin{array}{c} x_{\mathrm{ij}}^{\prime }=\frac{\max(x_{j})-x_{\mathrm{ij}}}{\max(x_{j})-\min(x_{j})} \end{array}$$

The processing yields a normalized matrix X′


(8) $$\begin{array}{c} X\prime =[\begin{array}{cccc} x_{11}\prime & x_{12}\prime & x_{13}\prime & x_{14}\prime \\ x_{21}\prime & x_{22}\prime & x_{23}\prime & x_{24}\prime \\ \vdots & \vdots & \vdots & \vdots \\ x_{m1}\prime & x_{m2}\prime & x_{m3}\prime & x_{m4}\prime \end{array}] \end{array}$$

Step 3: Calculation of indicator weights based on the entropy weight method.

(1) Calculate the proportion of j-th indicator of the i-th sample:


(9) $$\begin{array}{c} r_{\mathrm{ij}}=\frac{x_{\mathrm{ij}}\prime }{\sum_{i=1}^{m}x_{\mathrm{ij}}\prime } \end{array}$$

(2) Calculate the information entropy $e_{j}\;$ for the jth indicator:


(10) $$\begin{array}{c} e_{j}=-\frac{1}{\ln m}\sum_{i=1}^{m}r_{\mathrm{ij}}\ln r_{\mathrm{ij}} \end{array}$$

(3) Calculation of the indicator coefficient of variation $\;\;d_{j}$ :


(11) $$\begin{array}{c} d_{j}=1-e_{j} \end{array}$$

(4) Determine the entropy weight $w_{j}$ :


(12) $$\begin{array}{c} w_{j}=\frac{d_{j}}{\sum_{k=1}^{4}d_{k}} \end{array}$$

**Figure 2:**
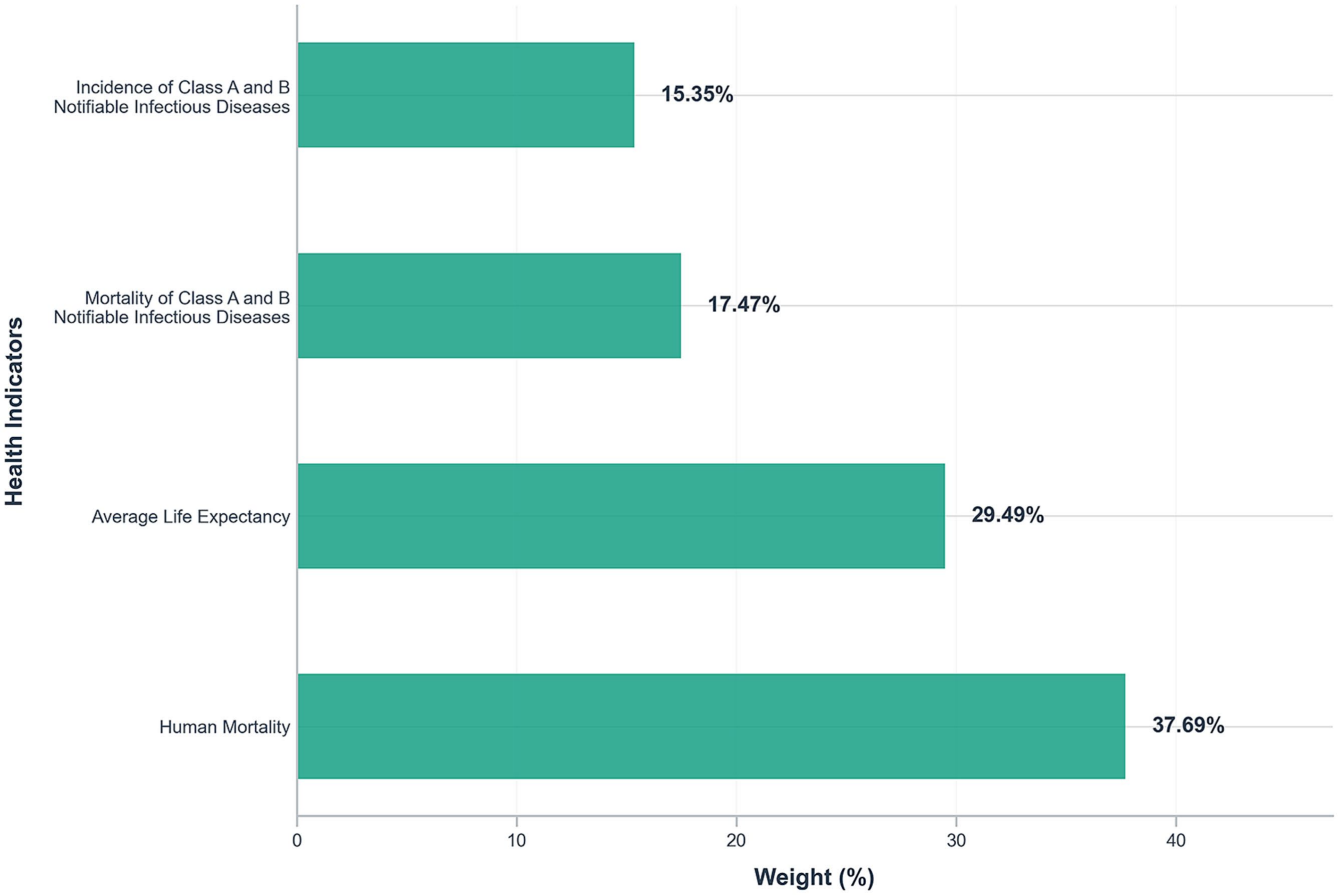
Weigh of health indicators.

The weight results are shown in Figure 2.

Step 4: TOPSIS method.

(1) Construct the weighted normalization matrix V:


(13) $$\begin{array}{c} \begin{array}{l} V=[\begin{array}{cccc} w_{1}x_{11}\prime & w_{2}x_{12}\prime & w_{3}x_{13}\prime & w_{4}x_{14}\prime \\ w_{1}x_{21}\prime & w_{2}x_{22}\prime & w_{3}x_{23}\prime & w_{4}x_{24}\prime \\ \vdots & \vdots & \vdots & \vdots \\ w_{1}x_{m1}\prime & w_{2}x_{m2}\prime & w_{3}x_{m3}\prime & w_{4}x_{m4}\prime \end{array}]\\ \;=[\begin{array}{cccc} v_{11} & v_{12} & v_{13} & v_{14}\\ v_{21} & v_{22} & v_{23} & v_{24}\\ \vdots & \vdots & \vdots & \vdots \\ v_{m1} & v_{m2} & v_{m3} & v_{m4} \end{array}] \end{array} \end{array}$$

(2) Determine the ideal solution:


(14) $$\begin{array}{c} \begin{array}{rr} & \;\;S_{j}^{+}=\max(v_{1j},v_{2j},\cdots ,v_{\mathrm{mj}})\\ & \;\;S_{j}^{-}=\min(v_{1j},v_{2j},\cdots ,v_{\mathrm{mj}}) \end{array} \end{array}$$

(3) Calculate the distance from the sample to the ideal solution:


(15) $$\begin{array}{c} \begin{array}{rr} & \;\;D_{i}^{+}=\sqrt{\sum_{j=1}^{4}{\left(v_{\mathrm{ij}}-S_{j}^{+}\right)}^{2}}\\ & \;\;D_{i}^{-}=\sqrt{\sum_{j=1}^{4}{\left(v_{\mathrm{ij}}-S_{j}^{-}\right)}^{2}} \end{array} \end{array}$$

(4) Calculate the closeness ${\mathrm{HLI}}_{i}$


(16) $$\begin{array}{c} {\mathrm{HLI}}_{i}=\frac{D_{i}^{-}}{D_{i}^{+}+D_{i}^{-}} \end{array}$$

${\mathrm{HLI}}_{i}\in [0,1]$ , with larger values indicating a higher health level of the province’s residents, and smaller values indicating a lower health level.

#### 2.2.2 XGBoost model

XGBoost (eXtreme Gradient Boosting) is an optimized implementation of the Gradient Boosting Decision Tree (GBDT) algorithm, which builds a strong learner by integrating multiple weak learners to achieve highly accurate predictions. The algorithm performs well in classification and regression tasks dealing with structured data, and is particularly suitable for machine learning scenarios with high feature dimensionality and large sample sizes (40)

XGBoost builds an additive model composed of K regression trees to predict an output ${\hat{y}}_{i}$ for each sample i . The model prediction is defined as


(17) $$\begin{array}{c} {\hat{y}}_{i}=\sum_{k=1}^{K}f_{k}(x_{i}),\;\;f_{k}\in F \end{array}$$

where $x_{i}\in {\mathbb{R}}^{d}$ is the input feature vector and ℱ is the space of regression trees. Each tree $f_{k}$ maps $x_{i}$ to a leaf score.

The objective function, minimized at the t -th boosting iteration, combines a convex loss function l and a regularization term Ω that controls tree complexity:


(18) $$\begin{array}{c} L^{\left(t\right)}=\sum_{i=1}^{n}l(y_{i},{\hat{y}}_{i}^{\left(t-1\right)}+f_{t}(x_{i}))+\Omega (f_{t}) \end{array}$$

Using a second-order Taylor expansion around ${\hat{y}}_{i}^{\left(t-1\right)}$ , the objective approximates to


(19) $$\begin{array}{c} {\tilde{L}}^{\left(t\right)}=\sum_{i=1}^{n}[g_{i}f_{t}(x_{i})+\frac{1}{2}h_{i}f_{t}^{2}(x_{i})]+\Omega (f_{t}) \end{array}$$

where $g_{i}=\partial_{{\hat{y}}_{i}^{\left(t-1\right)}}l(y_{i},{\hat{y}}_{i}^{\left(t-1\right)})$ and $h_{i}=\partial_{{\hat{y}}_{i}^{\left(t-1\right)}}^{2}l(y_{i},{\hat{y}}_{i}^{\left(t-1\right)})$ are the first and second derivatives of the loss. The regularization term typically includes the number of leaves T and leaf weights $w_{j}$ :


(20) $$\begin{array}{c} \Omega (f)=\mathrm{\gamma T}+\frac{1}{2}\lambda \sum_{j=1}^{T}w_{j}^{2} \end{array}$$

This allows efficient tree structure optimization via greedy split finding and leaf weight calculation.

In this study, the XGBoost model was implemented in Python. The optimal parameters were obtained using a random search method, taking into account the computational efficiency (41). The dataset was divided into training and test data at a ratio of 8:2 using a fixed random seed (seed = 2025) to ensure reproducibility, with resampling conducted at the province-year level to maintain temporal and spatial independence and avoid data leakage across different provinces and years. The K-fold cross-validation method was used (the value of K was 5 in this study). This method involves partitioning the training data into five equal subsets and iteratively using one subset for validation while training on the remaining four, a process repeated five times to generate a comprehensive assessment of the model’s generalization performance and effectively avoid the overfitting problem of the model presented during the training process. The finalized key parameters for the XGBoost model were set as follows: learning rate 0.05; n_estimators 200; max_depth 3.

#### 2.2.3 SHAP method

SHAP (SHapley Additive exPlanations) is based on the Shapley value theory from game theory, providing a unified interpretability framework for machine learning models (42). SHAP values provide a theoretically grounded measure of feature attribution by interpreting model output as an additive feature attribution:


(21) $$\begin{array}{c} {\hat{y}}_{i}=\phi_{0}+\sum_{j=1}^{d}\phi_{j}x_{\mathrm{ij}} \end{array}$$

Where $\phi_{0}=E[\hat{y}]$ is the expected model output, and $\phi_{j}$ quantifies the contribution of feature j to the prediction for instance i .

Formally, the SHAP value $\phi_{j}$ is calculated as follows:


(22) $$\begin{array}{c} \phi_{j}=\sum_{S\subseteq D\{j\}}\frac{|S|!(d-|S|-1)!}{d!}[f_{S\cup j}(x_{S\cup j})-f_{S}(x_{S})] \end{array}$$

where D={1,…,d} is the full feature set, S is a subset of features excluding j , and $f_{S}(x_{S})$ denotes the model output when only features in S are present. This weighted average of marginal contributions satisfies properties of local accuracy, consistency, and missingness, ensuring fair and interpretable explanations.

#### 2.2.4 Model evaluation method

This study uses three statistical metrics to evaluate model prediction performance: coefficient of determination (R^2^), root mean square error (RMSE), and mean absolute error (MAE). The specific formulas (Equations 23–25) are as follows:


(23) $$\begin{array}{c} R^{2}=1-\frac{\sum_{i=1}^{n}{\left(y_{i}-{\hat{y}}_{i}\right)}^{2}}{\sum_{i=1}^{n}{\left(y_{i}-\bar{y}\right)}^{2}} \end{array}$$

(24) $$\begin{array}{c} \text{RMSE}=\sqrt{\frac{1}{n}\sum_{i=1}^{n}{\left(y_{i}-{\hat{y}}_{i}\right)}^{2}} \end{array}$$

(25) $$\begin{array}{c} \mathrm{MAE}=\frac{1}{n}\sum_{i=1}^{n}|y_{i}-{\hat{y}}_{i}| \end{array}$$

Where:

$y_{i}$ represents the actual value for the i-th sample.

${\hat{y}}_{i}$ represents the predicted value for the i-th sample.

$\bar{y}$ represents the mean of all actual sample values.

n represents the total number of samples.

## 3 Results and analysis

### 3.1 Model performance comparison

**Figure 3:**
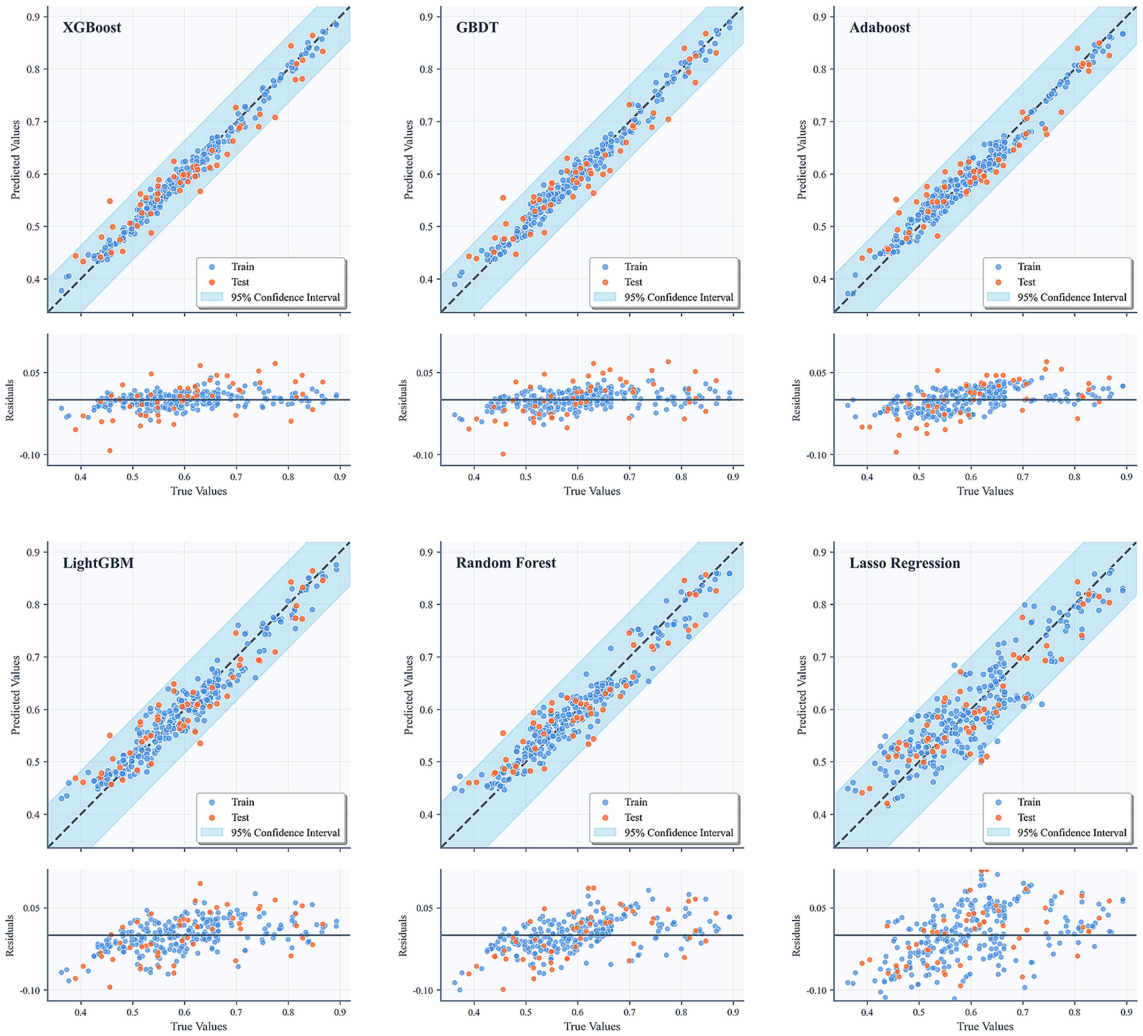
Performance of six models on the test and training sets.

In this study, six machine learning algorithms—XGBoost, AdaBoost, Gradient Boosting Decision Trees (GBDT), LightGBM, Random Forest, and Lasso regression—were systematically evaluated and compared in terms of their predictive performance. The detailed numerical results are presented in Table 4, while the corresponding residual plots and fitting performance plots are illustrated in Figure 3, providing a visual representation of the models’ predictive accuracy and residual distributions.

**Table 4:** Model performance comparison.

Model	Training set *R*^2^	Test set *R*^2^	Training set RMSE	Test set RMSE	Training set MAE	Test set MAE
XGBoost	0.988	0.929	0.012	0.033	0.009	0.026
GBDT	0.983	0.917	0.014	0.035	0.011	0.029
Adaboost	0.974	0.910	0.018	0.037	0.015	0.031
LightGBM	0.941	0.881	0.026	0.042	0.021	0.036
Random forest	0.932	0.871	0.029	0.044	0.022	0.038
Lasso regression	0.792	0.824	0.050	0.052	0.041	0.043

Overall, XGBoost demonstrated the most robust performance among all models across multiple evaluation metrics, highlighting its superior ability to capture complex, nonlinear relationships within the data. In particular, on the test set, XGBoost achieved a coefficient of determination (*R*^2^) of 0.929, which was not only substantially higher than that of the conventional linear regression approach (Lasso regression, *R*^2^ = 0.824) but also exceeded the performance of other ensemble-based methods such as AdaBoost (*R*^2^ = 0.910) and GBDT (*R*^2^ = 0.917). Furthermore, XGBoost attained the lowest root mean square error (RMSE = 0.033) and mean absolute error (MAE = 0.026) on the test dataset, underscoring its high predictive accuracy, minimal bias, and strong generalization capability.

The residual plots in Figure 3 further reinforce these findings, showing that the residuals of the XGBoost model are symmetrically distributed around zero, with no apparent heteroscedasticity or systematic patterns, indicating an adequate model fit and effective mitigation of overfitting. Similarly, the fitting performance plots depict strong linear correlations between the predicted and actual values for both the training and test sets, with the majority of points closely aligning with the 1:1 diagonal line, which represents perfect prediction. The inclusion of 95% confidence intervals provides an additional layer of interpretability, offering reasonable uncertainty bounds and further validating the reliability and robustness of the model’s predictions. Collectively, these results confirm that XGBoost not only delivers superior performance compared to traditional regression models and other ensemble methods but also maintains consistent accuracy and stability across different evaluation criteria, making it a compelling choice for predictive modeling in this context.

### 3.2 Feature importance analysis

**Figure 4:**
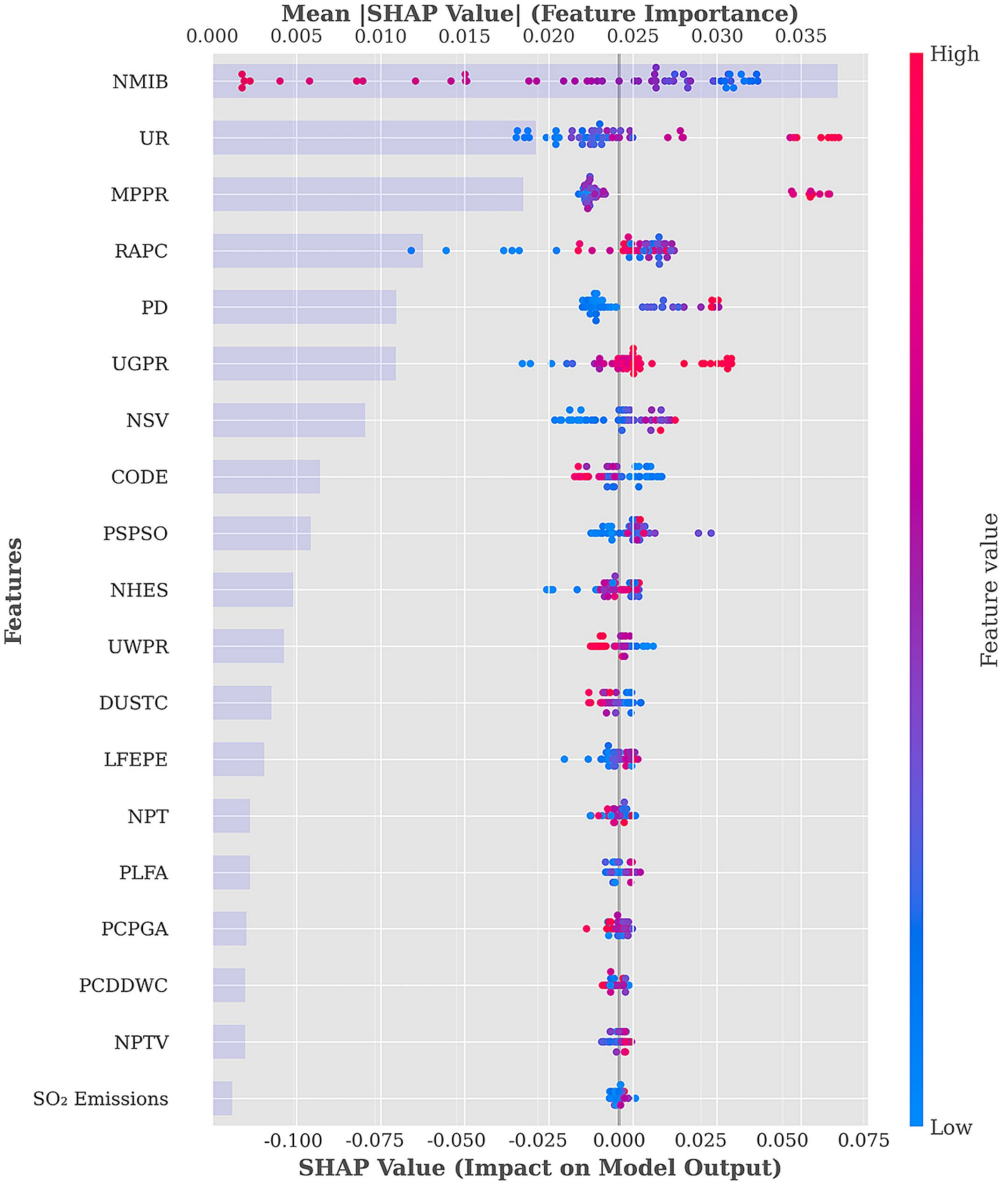
Feature summary and importance plot.

**Figure 5:**
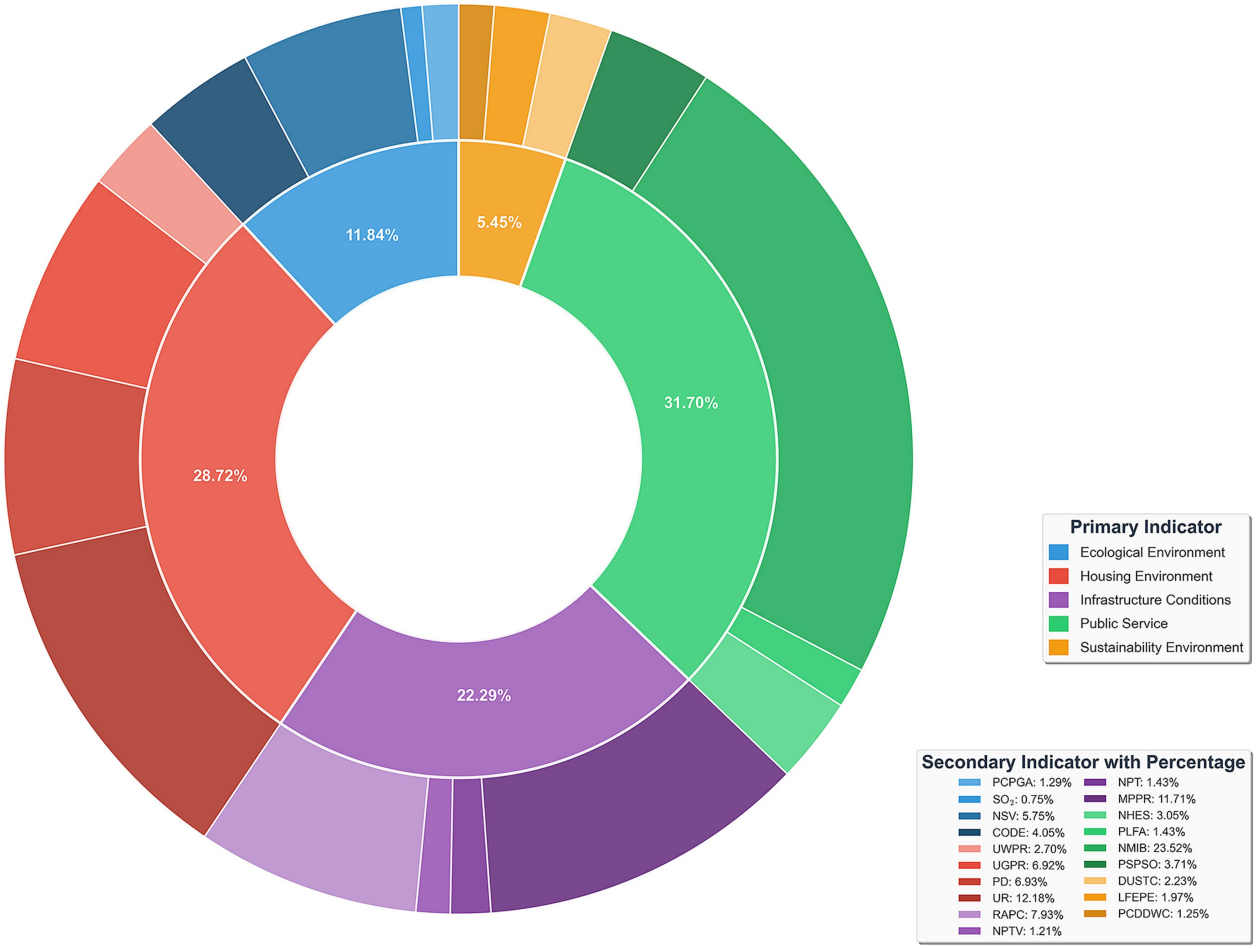
Percentage of feature importance.

SHAP values provide insights into both the direction (positive or negative) and the magnitude of each feature’s contribution to model predictions, offering a quantitative basis for identifying key influencing factors. Based on the SHAP mean absolute values, the importance ranking and distribution of 19 features in predicting the health level of the population are illustrated in Figure 4, while the percentage contribution of each feature is shown in Figure 5.

The SHAP summary plot and corresponding feature importance percentages reveal significant heterogeneity in the associations between various HSE features and population health. Among these, the top six most influential features— NMIB:23.52%, UR:12.18%, MPPR:11.71, RAPC:7.93%, PD:6.93%, and UGPR:6.92%—contribute substantially more than others, indicating their central role in reflecting health level.

**Figure 6:**
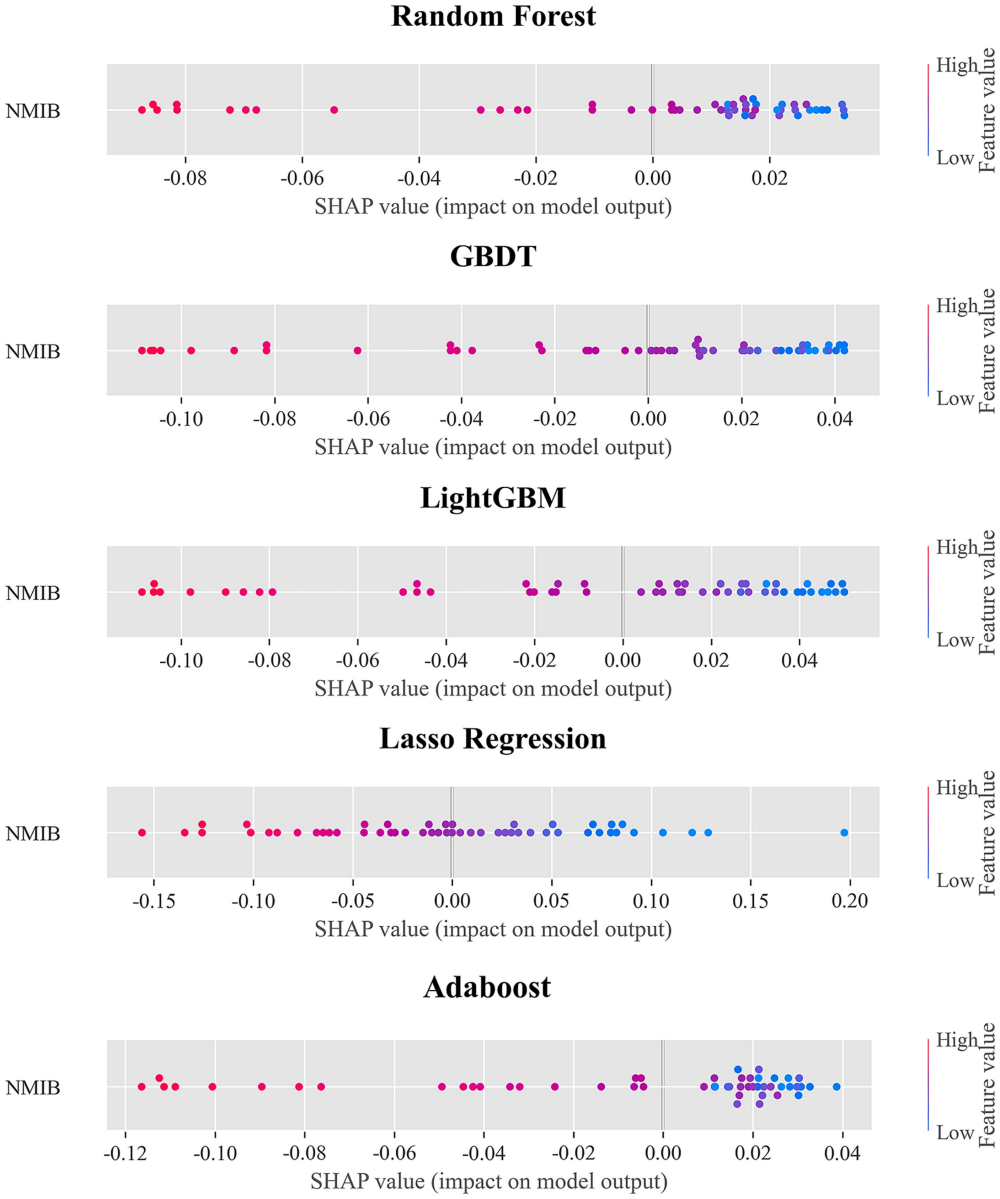
SHAP summary plot for NMIB in other models.

In the SHAP summary plot, NMIB displays a counterintuitive distribution: higher NMIB is predominantly associated with negative SHAP values, whereas lower NMIB corresponds more frequently with positive SHAP values. This pattern is consistently observed across the other five models, as shown in Figure 6, providing further support for our finding. From the perspective of health demand, the number of hospital beds reflects the health status and healthcare needs of local populations. Regions with a high NMIB often experience greater disease burdens, higher prevalence of chronic illnesses, or more advanced population aging. These areas require more hospital beds to meet elevated inpatient service demands. Consequently, a high NMIB may signal relatively poor population health level—an observation similar to prior findings (43, 44). From a healthcare service model perspective, modern systems increasingly prioritize disease prevention and outpatient care, aiming to reduce unnecessary hospitalizations. Healthier regions tend to have more robust public health infrastructures and advanced medical technologies, enabling effective prevention and outpatient management, which in turn reduces inpatient demand. Thus, such regions may exhibit lower NMIB while maintaining a higher overall health level. Population structure is another relevant factor. Areas with older populations—who typically require more frequent inpatient care—demand greater hospital bed capacity. Conversely, younger populations have lower hospitalization needs, reflected in lower NMIB. This ‘demand response effect’ suggests that the evaluation of healthcare resource allocation should consider health needs and demographic structures rather than assuming ‘more is better’. Optimal resource distribution should ensure basic needs are met while improving efficiency and outcomes through smarter planning and service innovation (45).

UR demonstrates a clear gradient in the SHAP value distribution, transitioning from negative SHAP values at low urbanization levels to positive values at higher levels. This pattern reflects the progressive health-promoting association of urbanization. Urbanized areas typically benefit from more comprehensive healthcare facilities and public service systems. Urbanization also brings improved sanitation infrastructure—such as water supply, sewage, and waste management—significantly enhancing living conditions and health security. As noted by Ngounou, Oumbe (46), urbanization also positively affects education. Residents in highly urbanized areas are more likely to access health education, disease prevention information, and modern health concepts. Enhanced health literacy fosters healthier lifestyles, greater self-care awareness, and better disease prevention, contributing to long-term health improvements. Urban environments also offer cultural, recreational, and fitness facilities that promote physical and mental well-being (47).

MPPR exhibits a clear polarization in its SHAP value distribution: low MPPR clusters around negative SHAP values, while high MPPR align with positive values. This underscores the role of the digital divide in health. Improved mobile phone penetration significantly enhances residents’ ability to access health-related information (48). In areas with high MPPR, residents can easily obtain disease prevention guidance, health behavior tips, and medical service information, contributing directly to better health literacy and behavioral improvements. In underserved regions, mobile internet acts as a vital supplement to traditional health education. From a healthcare access perspective, MPPR supports the development of digital healthcare services (49). The proliferation of telemedicine, online consultations, e-prescriptions, and mobile health apps helps mitigate healthcare resource imbalances, particularly enhancing service accessibility in under-resourced areas. Socially, mobile phones facilitate community engagement and social integration. Digital platforms enable participation in local activities, access to social support, and maintenance of interpersonal relationships—all of which are beneficial to mental health and overall well-being. However, it is important to acknowledge the potential downsides of mobile technology (50). In areas with low MPPR, limited information access and service availability may hinder health improvement, reflecting the adverse effects of the digital divide.

RAPC demonstrates a complex, nonlinear pattern in the SHAP value distribution. High RAPC values appear at both positive and negative values of the SHAP value spectrum, while moderate values tend to cluster around positive SHAP values. This indicates the multifaceted associations between transport infrastructure and health level. Access to transportation is closely linked to healthcare accessibility (51). Efficient transportation networks reduce commuting burden and emergency response times. In medical emergencies, accessible roads improve ambulance response and increase survival rates. However, excessive road infrastructure may also lead to adverse health outcomes. Densely developed road networks can increase traffic volume, air pollution, noise, and accident risks (52–54). In urban cores, expanded road areas may reduce green space and public recreational areas, diminishing environmental livability. In addition, overreliance on motorized transport may discourage physical activity such as walking or cycling, negatively impacting physical and mental health (55). The nonlinear effects of RAPC emphasize the importance of urban planning. Health-optimized transport infrastructure should balance mobility, environmental quality, and quality of life through better network design, investment in public transport, and the promotion of green mobility.

PD exhibits a clear gradient in SHAP value distribution: low PD is associated with negative SHAP values, while high PD is associated with positive values. This pattern suggests that moderate population agglomeration contributes positively to health levels. From a public service economy of scale perspective, higher PD facilitates more efficient allocation of healthcare, education, and cultural resources. Densely populated areas often offer more specialized and diverse services, including segmented healthcare services, better educational access, and greater availability of sports and cultural amenities (56). From the perspective of social support networks, higher population density is significantly associated with an increase in social support (57). In communities with relatively concentrated populations, social interactions among residents are more frequent, community cohesion is stronger, and an effective social support system can be formed. This kind of social support network plays a significant role in maintaining mental health, disease prevention, and promoting healthy behaviors. However, excessive population density may result in environmental degradation (58). Thus, the analysis highlights the positive associations between moderate population agglomeration and health level rather than uniformly endorsing high-density development.

UGPR, an important indicator of clean energy adoption, shows a distinctly positive association with health level in the SHAP analysis. High UGPR values correspond to positive SHAP values, whereas low UGPR is associated with negative SHAP values, underscoring the significance of energy structure optimization for public health. Gas coverage’s contribution to health predictions is primarily associated with improvements in air quality. Compared to traditional coal combustion, natural gas—being a cleaner fossil fuel—generates significantly fewer pollutants such as particulate matter, sulfur dioxide, and nitrogen oxides (59). Regions with high gas penetration have lower air pollution emissions and better air quality, thereby reducing the incidence of respiratory diseases. This is particularly important in northern regions where gas has replaced coal for winter heating. From an indoor air quality perspective, widespread gas use significantly improves the domestic environment. Traditional coal-based heating and cooking generate substantial indoor pollutants such as carbon monoxide and sulfur dioxide. Clean gas combustion reduces these risks dramatically (60), lowering residents’ exposure to hazardous indoor air. UGPR also reflects the modernization of urban infrastructure. High gas coverage requires comprehensive pipeline systems, safety protocols, and user-friendly service mechanisms, indicating advanced urban governance and public service quality.

### 3.3 Non-linear relationship and threshold effects analysis

**Figure 7:**
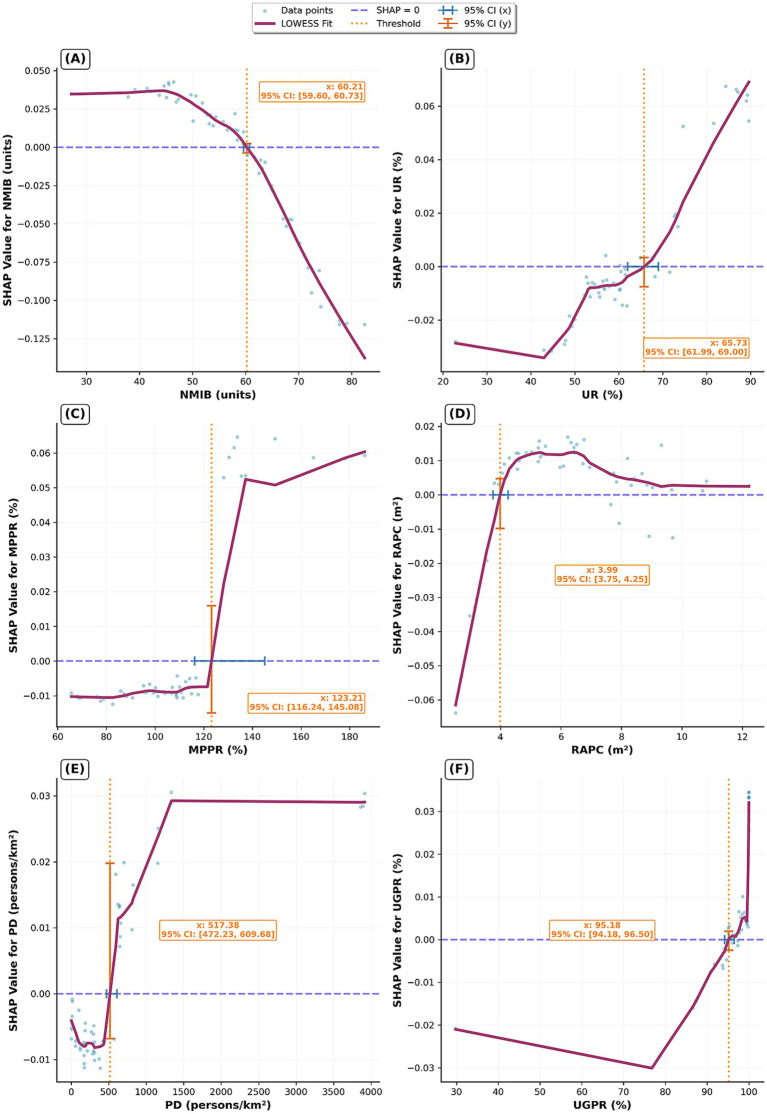
Nonlinear impacts and threshold effects of the HSE on HLI. **(A)** NMIB; **(B)** UR; **(C)** MPPR; **(D)** RAPC; **(E)** PD; **(F)** UGPR.

To elucidate the complex nonlinear associations and potential threshold effects between the top six important features and HLI, this study employed locally weighted scatterplot smoothing (LOWESS) on SHAP value scatter plots to derive smoothed fitting curves. A smoothing span of 0.3 was determined through iterative experimentation and visual evaluation to balance the risks of overfitting and underfitting. A locally linear fitting method was adopted to enhance the robustness of the estimation. The threshold for each feature was defined as the point on the horizontal axis at which the fitted curve intersected the reference line corresponding to a SHAP value of zero. The uncertainty associated with these thresholds was quantified by constructing 95% confidence intervals using a bootstrap resampling procedure. The plots are shown in Figure 7.

Figure 7A reveals a distinct nonlinear relationship between NMIB and population health level, with a critical turning point at 60.21 (95% CI: 59.60–60.73) beds per 10,000 people. Below this threshold, increases in NMIB are associated with positive SHAP values, indicating that a higher availability of hospital beds contributes significantly to better health levels. This aligns with expectations, as adequate inpatient capacity ensures timely and effective treatment, thereby reducing morbidity and mortality rates. However, once NMIB exceeds approximately 60 beds per 10,000, SHAP values become negative. This phenomenon may reflect inefficient utilization of medical resources in certain provinces (61), or it may signal more severe public health challenges that require disproportionately greater healthcare infrastructure to manage.

Figure 7B illustrates the complex associations between urbanization and population health level, with a threshold at 65.73% (95% CI: 61.99–69.00). At a low urbanization level, SHAP values are negative, suggesting that early stages of urbanization are associated with a poorer health level. This may be correlated with increased environmental pollution, lifestyle shifts, and heightened social stress associated with rapid urban transition. Importantly, this negative association diminishes as urbanization progresses. After surpassing the threshold range, the SHAP values turn positive, indicating a reversal in the association. At this stage, the benefits of urban development—such as centralized medical resources, improved infrastructure, and higher educational attainment—begin to outweigh earlier disadvantages. Highly urbanized areas typically offer superior health systems, robust public health infrastructure, and greater health awareness, collectively contributing to improved population health levels. Overall, urbanization has played a positive role in the health level of the population, which is consistent with previous studies (62, 63).

Figure 7C identifies a distinct threshold effect at 123.21 (95% CI: 116.24–145.08) units/100 persons. Below this level, the association between increasing MPPR and health level is limited, with SHAP values remaining relatively low. However, once MPPR exceeds this threshold, its positive contribution to health predictions becomes pronounced and stabilizes. This suggests a digital threshold pattern: when mobile connectivity reaches a certain saturation point, digital health services—such as telemedicine, health monitoring, and access to medical information—become widespread. The availability of these services is associated with improved healthcare accessibility and efficiency.

Figure 7D highlights an early threshold effect for RAPC at 3.99 (95% CI: 3.75–4.25) m^2^ per person. Below this level, increases in RAPC are associated with negative SHAP values, possibly due to negative externalities such as pollution, noise, and disruptions linked to early-stage road construction. Once RAPC surpasses the threshold range, the relationship turns positive and remains relatively stable. This indicates that a certain level of transport infrastructure improves access to healthcare services and supports health-related mobility. However, it also underscores the need to balance improved accessibility with potential environmental and social costs of overdevelopment.

Figure 7E reveals a threshold effect of PD on health, with a key turning point at 517.38 (95% CI: 472.23–609.68) persons/km^2^, after which the health level increases until reaching a plateau at approximately 1,250 persons/km^2^. In the low-density phase, increases in PD slightly reduce health level, as reflected in negative SHAP values. Beyond the threshold range, the association turns significantly positive, indicating that economies of scale associated with population concentration begin to show positive correlations with health level. Moderate population density facilitates efficient distribution of healthcare resources, scaled public health services, and stronger social networks. At around 1,250 persons/km^2^, the fitted curve plateaus, and SHAP values stabilize at a high level, indicating a diminishing marginal effect of PD on health. This suggests an optimal density range where health benefits from population agglomeration. Several mechanisms may explain this plateau effect. Firstly, the allocation of medical resources and the public health service system in high PD areas are already relatively well-established. Further increases in population density are unlikely to yield significant marginal improvements in public health levels. Secondly, excessive PD may give rise to a range of negative factors, including intensified environmental pollution (64), heightened perception of stress (65), and an increased risk of infectious disease transmission (66). These adverse effects may offset some of the health benefits associated with population agglomeration.

Figure 7F reveals a high threshold of UGPR at 95.18 (95% CI: 94.18–96.50) %. Before reaching this threshold, UGPR has a slight negative trend in SHAP values. However, once gas penetration exceeds the threshold range, its contribution to health predictions sharply turns positive. This inflection likely reflects network and quality effects of gas infrastructure.

### 3.4 Analysis of interaction effects among HSE features

**Figure 8:**
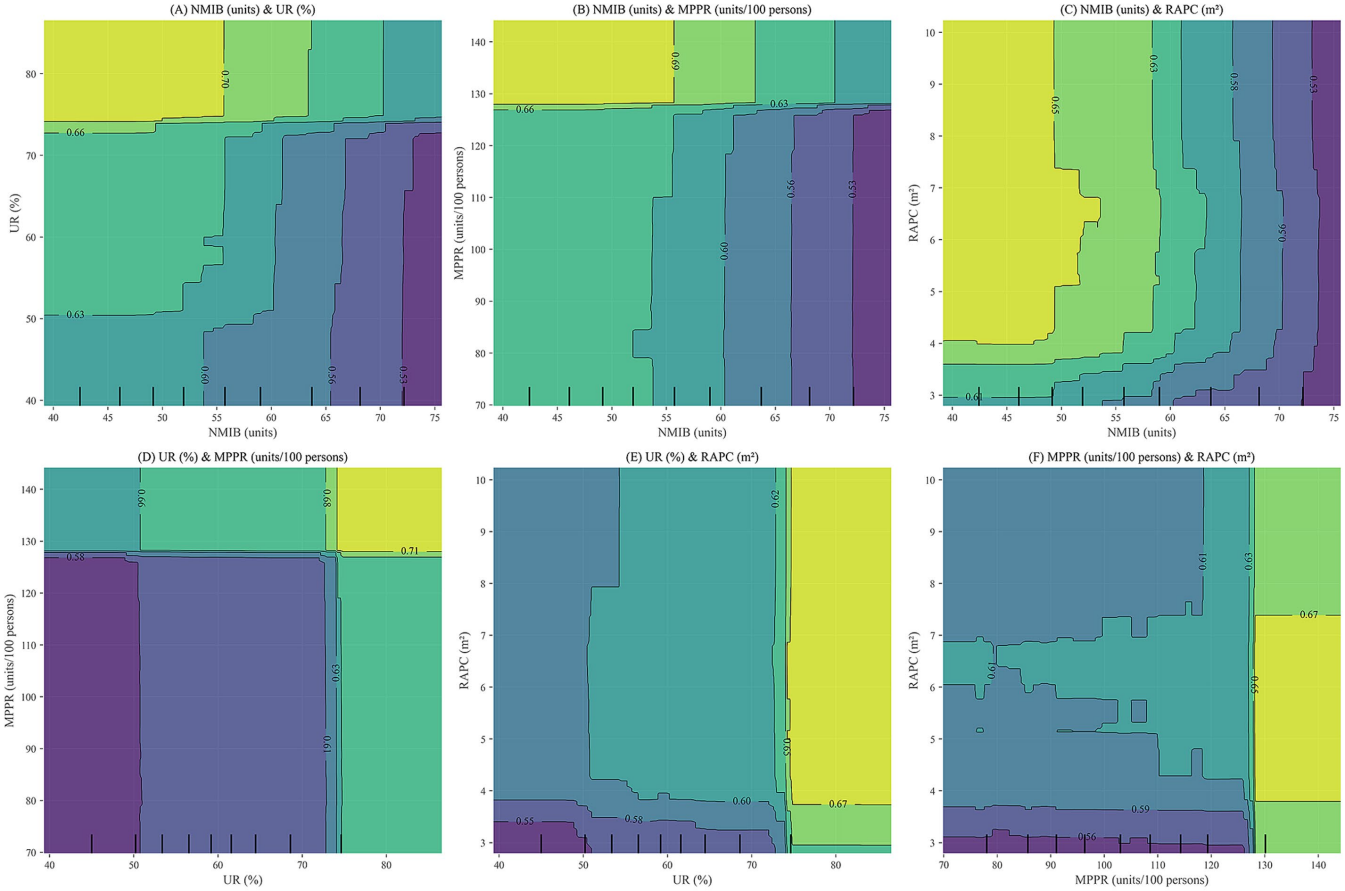
PDP plots for NMIB, UR, MPPR, and RAPC. **(A)** NMIB & UR; **(B)** NMIB & MPPR; **(C)** NMIB & RAPC; **(D)** UR & MPPR; **(E)** UR & RAPC; **(F)** MPPR & RAPC.

The PDP plots (Figure 8) reveal complex interactive effects of NMIB, UR, MPPR, and RAPC on HLI. High HLI values are typically observed under conditions of high UR, high MPPR, and high RAPC. However, NMIB does not exhibit a simple positive relationship with HLI; instead, a reverse association is identified.

Specifically, in regions with relatively low NMIB but high UR (Figure 8A), HLI tends to be higher. This suggests that urbanization is associated with higher population health levels, potentially linked to improved living conditions, healthcare accessibility, public health infrastructure, and healthier lifestyles. A lower NMIB may indicate reduced health pressure or more efficient medical resource utilization in these areas.

In Figure 8B, higher HLI values are concentrated in regions with high MPPR and moderate-to-low NMIB, implying that robust digital infrastructure is associated with more efficient health management and service delivery, which may reduce dependency on large numbers of hospital beds. Conversely, in areas with low MPPR, even with a high NMIB, HLI remains suboptimal. This indicates that merely expanding healthcare infrastructure is not necessarily associated with higher health levels without adequate digital support.

Figure 8C shows that high HLI values primarily occur in regions with high RAPC and moderate-to-low NMIB. This demonstrates that sufficient transportation infrastructure is associated with improved medical accessibility and emergency responsiveness, which may be linked to lower health risks and reduced reliance on hospital beds. In contrast, limited road infrastructure is associated with traffic congestion and delayed emergency response, potentially constraining the effectiveness of medical resources, regardless of bed availability.

Moreover, UR demonstrates significant synergistic interactions with MPPR and RAPC (Figures 8D–F). The combination of high UR and high MPPR is associated with higher HLI, potentially reflecting complementary effects of health information access and dissemination. Similarly, high UR paired with high RAPC is associated with higher health levels, potentially reflecting reduced urban congestion and medical service delays. The synergy between digitalization (MPPR) and physical infrastructure (RAPC) is also evident, where improvements in both simultaneously elevate the health level. High MPPR enhances the responsiveness and reach of health services, while high RAPC ensures spatial accessibility for medical resource allocation.

In summary, the PDP plots illustrate that NMIB does not simply reflect the adequacy of healthcare resources but also serves as a composite indicator of urban health risks, disease burden, and healthcare system efficiency. Improvements in HLI correlate with the coordinated contributions of multiple environmental factors. Solely expanding hospital capacity is not necessarily associated with higher health levels; it may reflect inefficiencies or reactive health governance. Optimal HLI levels are generally found in conditions characterized by high UR, strong digital infrastructure (MPPR), Well-developed infrastructure (RAPC), and moderate hospital bed supply. This highlights the importance of structural optimization and systemic synergy in urban health governance. Future health planning should focus on the integrated development of urbanization, informatization, and infrastructure while improving the operational efficiency of healthcare systems and rational allocation of hospital beds to establish a multidimensional, coupled framework for health governance.

## 4 Conclusion and discussion

This study deciphers the intricate relationship between HSE and provincial health level in China through a machine learning-driven analytical framework. The findings reveal that this relationship is neither linear nor additive; instead, it is characterized by complex nonlinearities, threshold effects, and synergistic interactions across multiple environmental dimensions.

A key insight of the analysis is the prominent role of six core indicators—NMIB, UR, MPPR, RAPC, PD, and UGPR—in predicting HLI. Contrary to conventional assumptions, a higher NMIB, typically viewed as a sign of enhanced healthcare capacity, does not necessarily correlate with better health. SHAP and PDP analyses indicate a dual implication of NMIB: it reflects both healthcare supply and potential system inefficiencies or underlying health burdens. Excessive bed supply may suggest either increased disease prevalence or misallocation of medical resources.

The LOWESS curves further reveal distinct nonlinear patterns and threshold effects. For instance, UR begins to exert a positive influence on health only after surpassing the threshold range. PD exhibits two critical points—517.38 (95% CI: 472.23–609.68) and approximately 1,250 persons/km^2^—indicating that moderate population agglomeration is associated with higher health levels, while excessively high densities are linked with diminishing or even negative health outcomes. Similarly, MPPR and UGPR only demonstrate significant positive associations with health level once high threshold levels are exceeded.

Interaction analysis based on PDP plots underscores that health improvements are not strongly associated with isolated environmental factors, but rather with the coordinated optimization of multiple HSE dimensions. The joint presence of high UR, high MPPR, and high RAPC is associated with higher health levels. Synergistic effects between urbanization and digital infrastructure, as well as between urbanization and physical infrastructure, are linked with higher health levels. These findings highlight the importance of integrated, system-level health governance that accounts for the interplay among various environmental components.

However, several limitations warrant attention. Although this study employs panel data (31 provinces over 11 years), the XGBoost model treats the data as pooled cross-sections, thus overlooking temporal dependencies and potential lagged effects. The model does not capture province-specific fixed effects, which may lead to biased estimations due to unobserved heterogeneity. While modeling temporal dynamics with tree-based methods remains challenging, future research could incorporate year dummies, lagged variables, or hybrid modeling techniques to better capture dynamic relationships.

Additionally, SHAP values provide transparent feature attribution but reflect statistical associations rather than causal mechanisms. As such, findings remain susceptible to unmeasured confounding. Readers should interpret the identified relationships—especially those involving complex variables such as NMIB, UR, MPPR, and RAPC—as correlational patterns, not definitive causal pathways.

Given China’s vast regional diversity, exploring the heterogeneity of HSE–health relationships across different regions (e.g., eastern vs. western, urban vs. rural) could enhance the policy relevance of the findings. While this study does not conduct a stratified regional analysis due to data and methodological constraints, future research should explicitly address regional variation to better tailor policy recommendations. This limitation should be acknowledged as an avenue for future exploration.

Finally, the use of provincial-level data, while effective in capturing macro-level trends, obscures intra-urban and individual-level variation. For example, disparities between urban neighborhoods or vulnerable population subgroups remain hidden. The HSE indicator system, though comprehensive across five dimensions, is constrained by data availability. It omits critical factors such as indoor environmental quality, housing conditions, mental health status, and subjective well-being—all of which are essential for a more holistic understanding of health.

Policy implications derived from this study offer valuable directions for improving health levels through more nuanced environmental and infrastructural planning. The findings underscore that health is shaped not by isolated environmental indicators but through complex, nonlinear interactions and threshold effects across multiple dimensions of HSE. Therefore, health-oriented policy should move beyond one-size-fits-all approaches and instead embrace integrated, system-level governance. For instance, coordinated investments in urbanization, digital infrastructure, and transportation systems can generate synergistic effects that substantially improve public health, particularly when these dimensions are jointly optimized. This has important implications for cross-sectoral planning—urban development, healthcare, technology, and transportation must be aligned to maximize health returns. Moreover, these insights can inform region-specific policy strategies. Provinces with a lower level of urbanization or digital infrastructure should prioritize foundational investments, such as expanding access to primary healthcare facilities, improving road connectivity, and enhancing digital inclusion. In contrast, highly urbanized regions—especially those approaching or exceeding population density thresholds—should focus on managing urban congestion, mitigating pollution, and optimizing the distribution of healthcare resources to avoid inefficiencies or over-concentration. It is also crucial to recognize potential trade-offs. For example, excessive expansion of urban infrastructure without proper environmental safeguards may lead to resource misallocation, ecological degradation, or increased social inequality. Policymakers must therefore weigh short-term development gains against long-term health and sustainability outcomes.

Building upon the current findings, future research should aim to address several key limitations and deepen the understanding of the HSE–health nexus. First, methodological improvements are needed to better capture the temporal dynamics and fixed effects inherent in panel data. Incorporating lagged variables, time dummies, or adopting hybrid models that integrate tree-based algorithms with panel regression techniques may enhance causal inference and temporal sensitivity. Second, future studies should strive to mitigate unmeasured confounding by expanding the scope of environmental and health indicators. This includes integrating variables such as indoor air quality, housing conditions, noise exposure, green space accessibility, and subjective well-being measures—factors currently absent due to data constraints but essential for a more holistic health assessment.

Moreover, future research should explore regional heterogeneity by conducting stratified analyses across geographic and socioeconomic divisions (e.g., eastern vs. western provinces, urban vs. rural areas). Such analysis would provide more context-sensitive insights and support differentiated policy interventions. Multi-scale data integration—linking provincial, municipal, neighborhood, and individual-level datasets—should also be prioritized. The use of high-resolution environmental data from remote sensing, geospatial platforms, and community health surveys can significantly enhance spatial granularity and policy relevance.

Lastly, incorporating behavioral and psychosocial health dimensions—such as physical activity, dietary habits, and mental health status—will further enrich the analytical framework. These enhancements will collectively support the development of more targeted, equitable, and sustainable health and urban planning strategies in diverse settings.

## Data Availability

Publicly available datasets were analyzed in this study. This data can be found here: https://www.jianguoyun.com/p/DQclQNkQ59HLDRjg84MGIAA.
