# Supplementary figures and images for: Decoding the association between health level and human settlements environment: a machine learning-driven provincial analysis in China
Source: Front Public Health. 2025 Sep 3;13:1672479. doi: 10.3389/fpubh.2025.1672479 (PMC12440893; doi:10.3389/fpubh.2025.1672479)

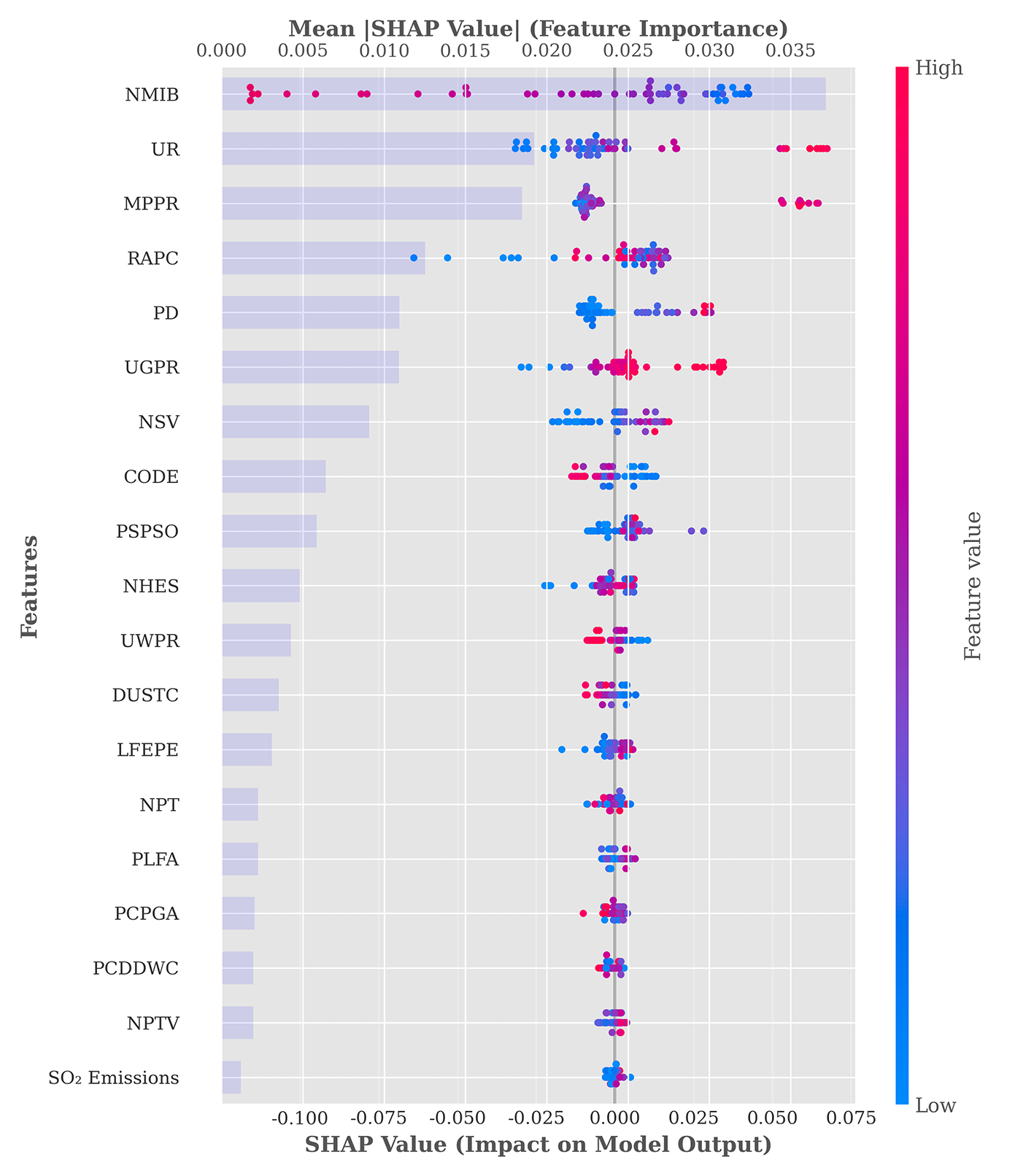

Supplement: Supplementary file 1 [file Data_Sheet_1.ZIP › Figures/Figure 2.tif]

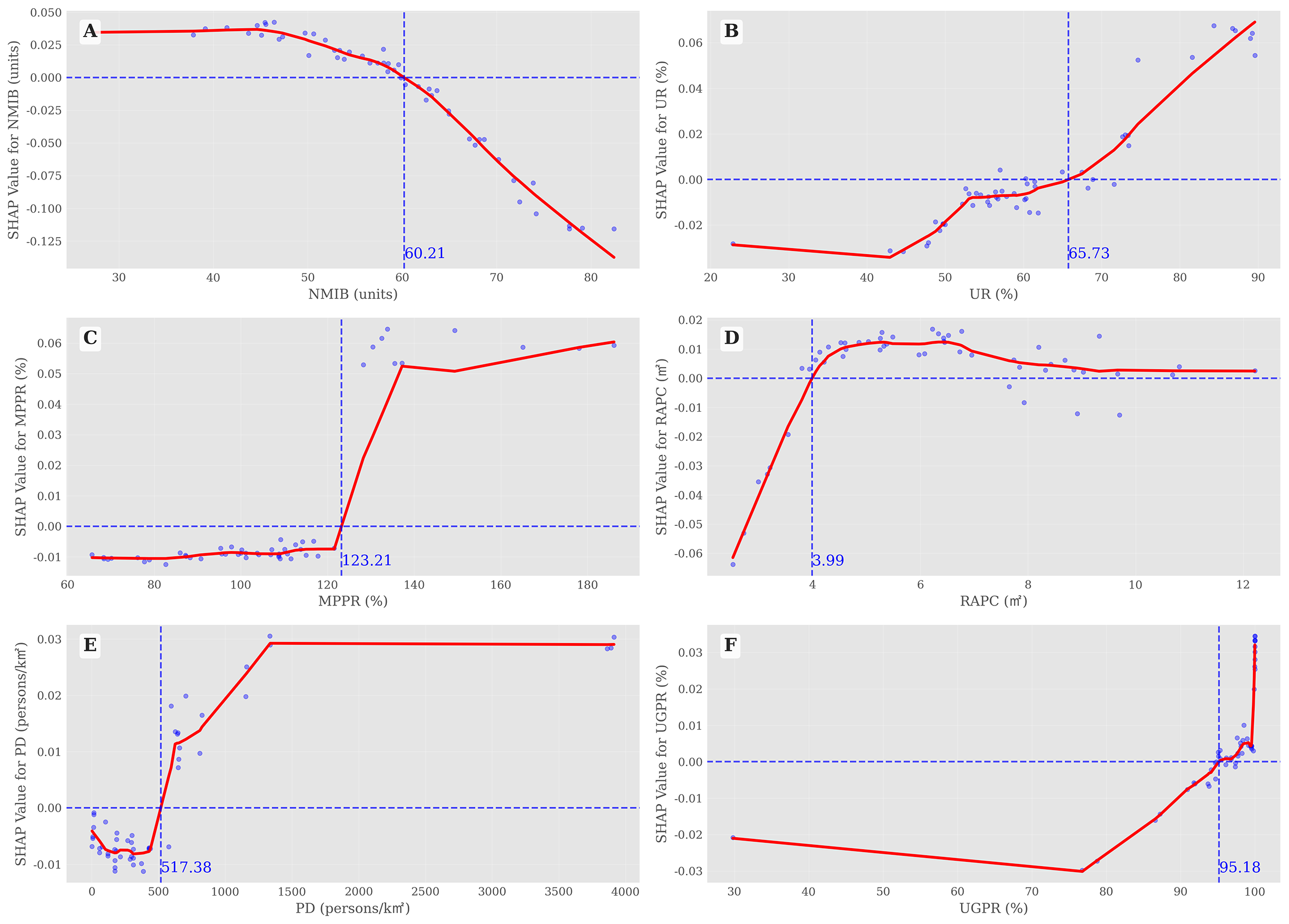

Supplement: Supplementary file 1 [file Data_Sheet_1.ZIP › Figures/Figure 4.tif]

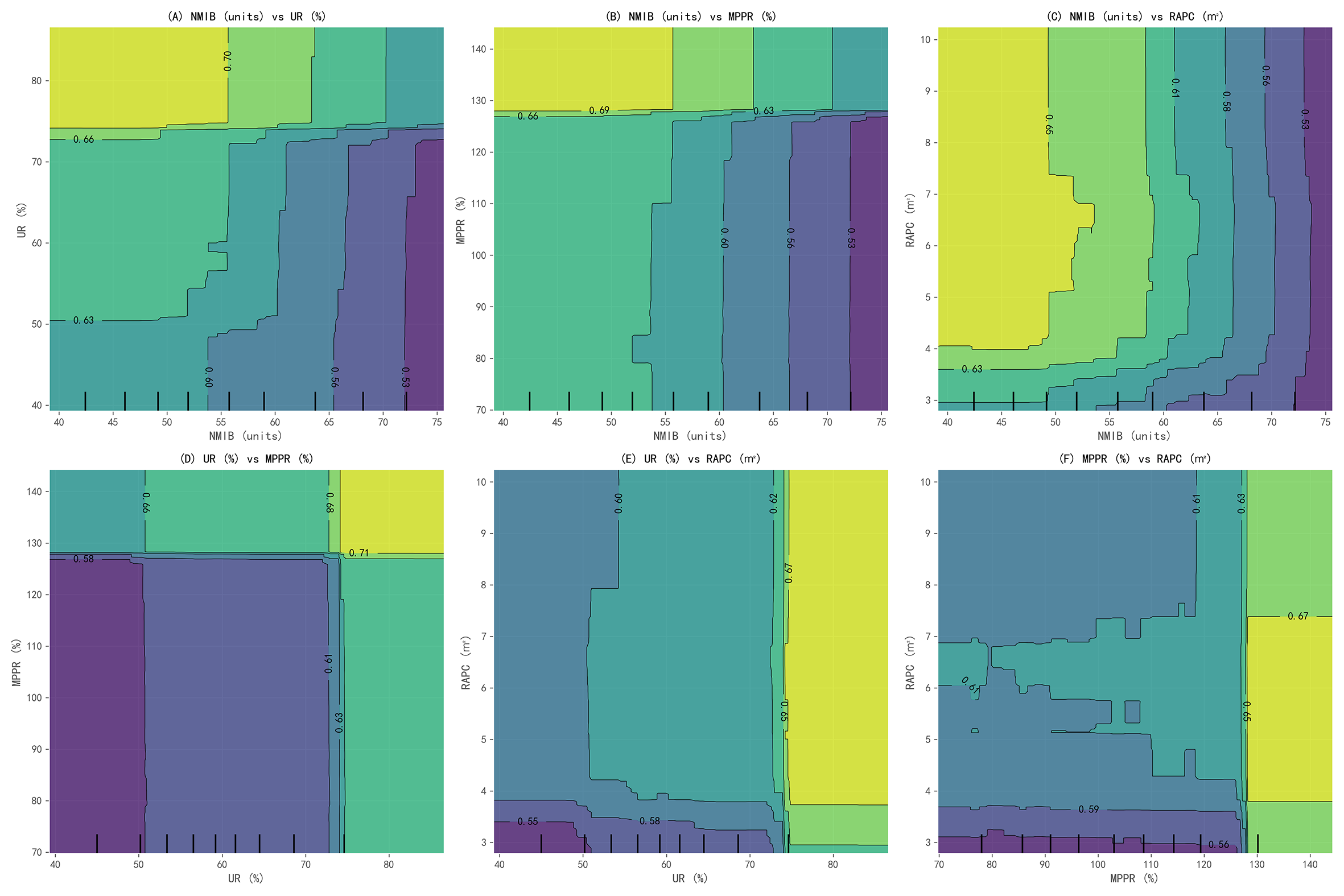

Supplement: Supplementary file 1 [file Data_Sheet_1.ZIP › Figures/Figure 5.tif]

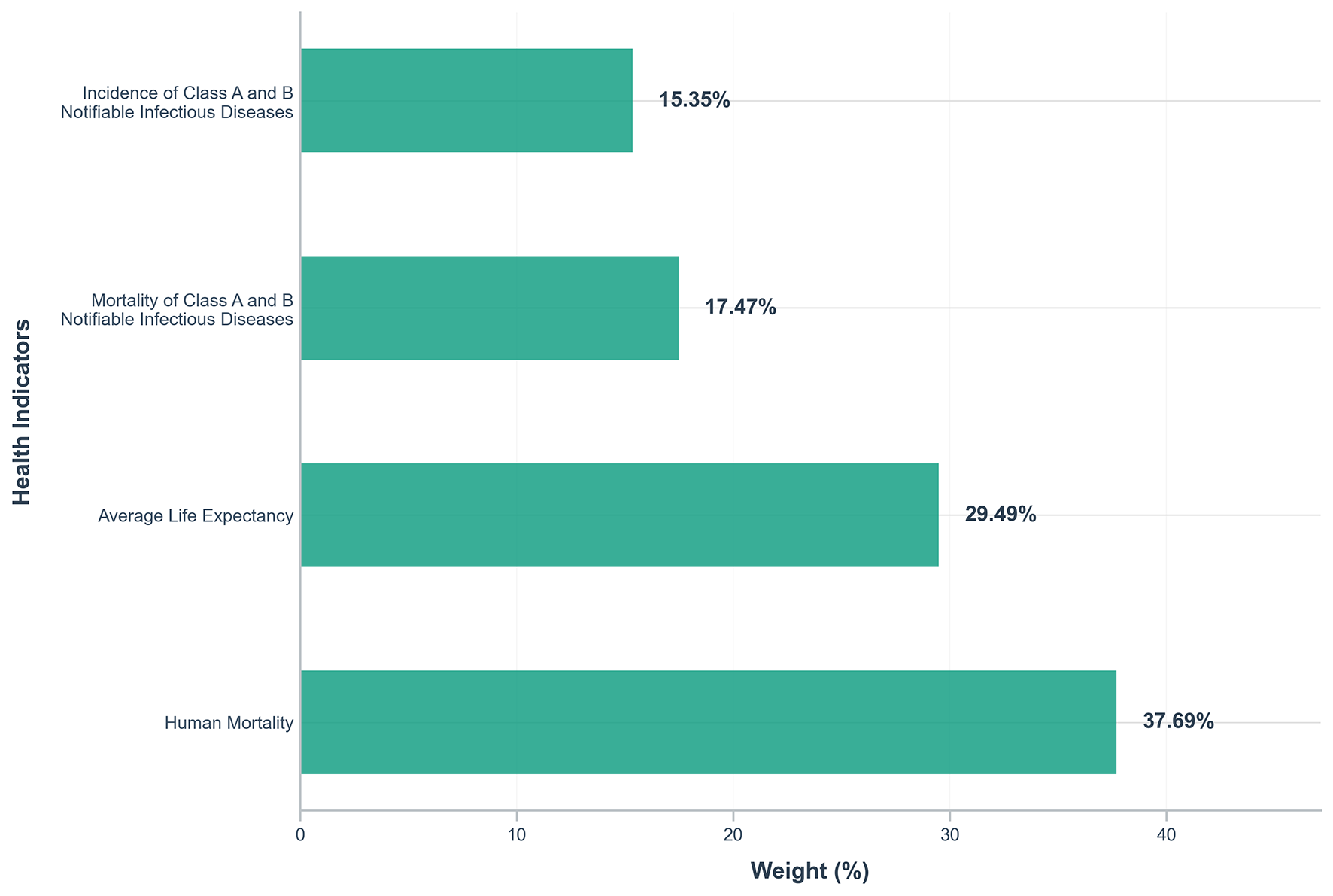

Supplement: Supplementary file 1 [file Data_Sheet_1.ZIP › Figures/Figure1.tif]

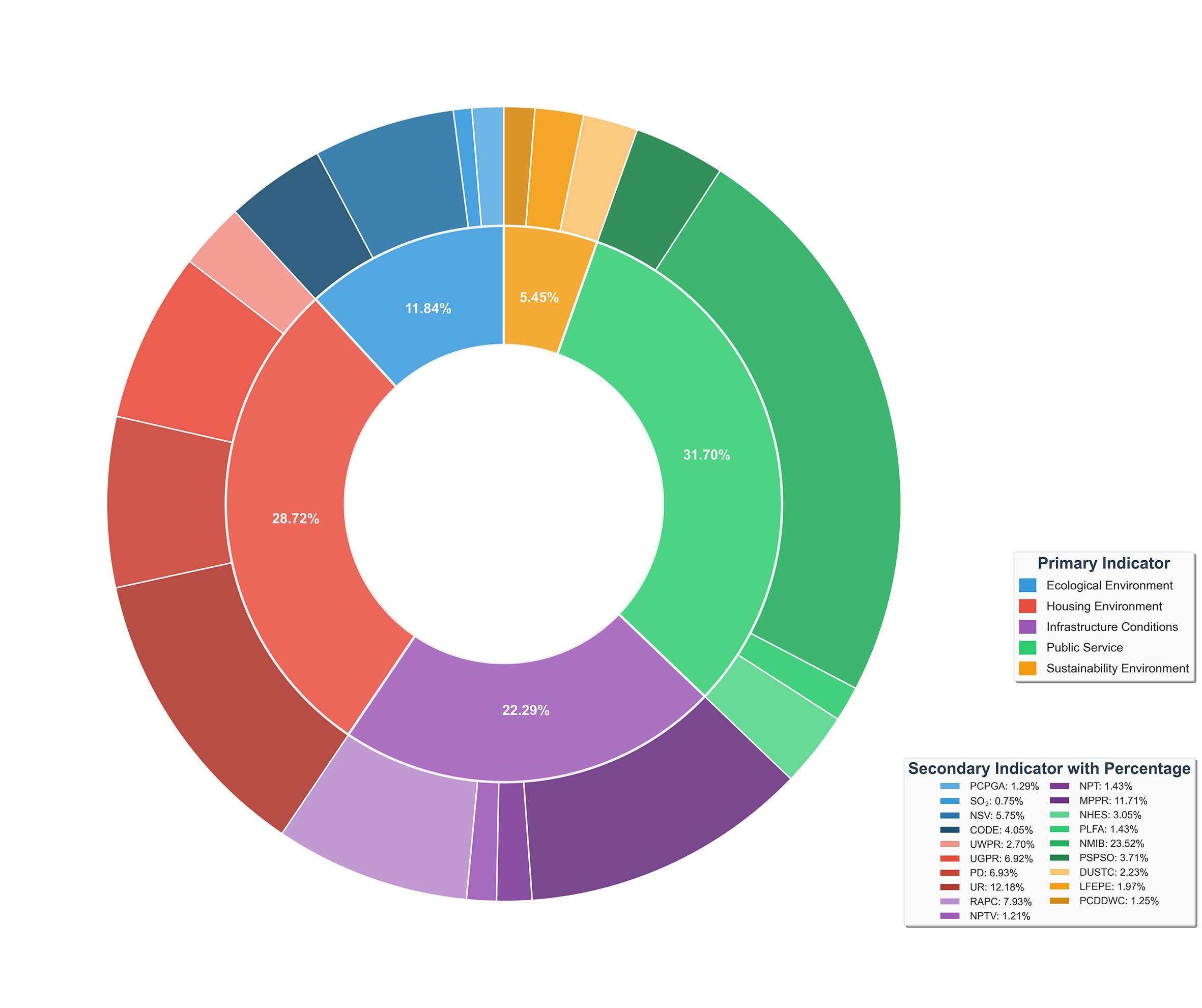

Supplement: Supplementary file 1 [file Data_Sheet_1.ZIP › Figures/Figure3.tif]
